# The Ability of Two Chewing Simulation Devices in Emulating the Clinical Deterioration of Anterior Composite Restorations in Severely Worn Teeth

**DOI:** 10.3290/j.jad.b2701665

**Published:** 2022-03-01

**Authors:** Verônica P. Lima, Rafael R. Moraes, Niek J.M. Opdam, Jan L. Ruben, Marie-Charlotte D.N.J.M. Huysmans, Bas A.C. Loomans

**Affiliations:** a PhD Candidate, Graduate Program in Dentistry, Federal University of Pelotas, Pelotas, RS, Brazil; PhD candidate, Radboud University Medical Center, Radboud Institute for Health Sciences, Department of Dentistry, Nijmegen, The Netherlands. Conceptualization, methodology, writing, editing, and critical review of manuscript.; b Professor, Graduate Program in Dentistry, Federal University of Pelotas, Pelotas, RS, Brazil. Conceptualization, methodology, writing, editing, supervision of experiments, drafting and critical review of manuscript.; c Associate Professor, Radboud University Medical Center, Radboud Institute for Health Sciences, Department of Dentistry, Nijmegen, The Netherlands. Conceptualization and critical review of manuscript.; d Research Analyst, Radboud University Medical Center, Radboud Institute for Health Sciences, Department of Dentistry, Nijmegen, The Netherlands. Methodology and critical review of manuscript.; e Professor, Radboud University Medical Center, Radboud Institute for Health Sciences, Department of Dentistry, Nijmegen, The Netherlands. Conceptualization and critical review of manuscript.; f Professor, Radboud University Medical Center, Radboud Institute for Health Sciences, Department of Dentistry, Nijmegen, The Netherlands. Conceptualization, methodology, writing, editing, and critical review of the manuscript.

**Keywords:** dental restoration, degradation, tooth wear, chewing simulation methods, FDI scores

## Abstract

**Purpose:**

This study investigated the ability of two chewing simulation devices to emulate in vitro the clinical deterioration observed in anterior composite restorations in severe tooth-wear patients.

**Materials and Methods:**

Advanced tooth wear was simulated in bovine incisors, which were restored with palatal and buccal direct composite veneer restorations. The incisal edges of restorations were subjected to 960K cycles of either compressive loading (Biocycle-V2; 125 N at 2 Hz) or wear and mechanical loading (Rub&Roll; 30 N at 20 rpm). Surface degradation was rated using FDI scores to compare the chewing devices (Fisher’s test, α = 0.05). Topography and deterioration of restorations was analyzed using SEM. The ability to emulate the deterioration was investigated by comparing the surface degradation observed in vitro with the clinical degradation observed in restorations placed in severe tooth-wear patients after 3.5 years.

**Results:**

Distinct degradation patterns were observed between the simulation devices: Biocycle-V2 generated deterioration that was not comparable to the clinical situation, including contact damage, minor wear, and localized roughening. The degradation caused by Rub&Roll was more similar to the in vivo situation, including wear facets, chipping, delamination, staining, and marginal ditching. The FDI scores were different between the chewing devices for surface/marginal staining, material/retention, and marginal adaptation (p ≤ 0.003). SEM analysis showed microcracking at the interface between composite layers at the incisal edges.

**Conclusions:**

The Rub&Roll chewing device was able to emulate the clinical deterioration observed in anterior restorations in severe tooth-wear patients and thus may be used as an oral-cavity simulation method, contributing to translational research.

Tooth wear is a multifactorial condition leading to irreversible loss of dental hard tissues, with an estimated global prevalence between 20% and 45% in the permanent dentition.^43^ The condition is considered severe when more than one-third of the clinical crown has been lost, a situation that is a restorative challenge.^30^ Restorative treatment should be minimally invasive and the restorations mostly additive, because patients have already experienced accentuated dental tissue loss. Clinical evidence suggests that resin composites are appropriate materials to restore worn teeth,^13,19,31,32^ with the advantage of being easily repairable.

Severe tooth-wear patients represent an unusual group of patients, as the etiological factors will most likely still be present after any restorative treatment is performed. Restorations will not necessarily stop nor prevent the wear processes, but they can alter the pace, site, and nature of wear.^30^ Clinical studies^4,13,14,19,31,37,45,47^ have reported that anterior resin composite restorations placed in those patients exhibit some distinctive features over time, namely, wear facets, marginal step or irregularities, staining, discoloration, chipping, and bulk fractures.^24^ Wear facets can be present even at early stages after the placement of restorations, and they are characterized by a large worn surface area or an inclined contact area with the opposing dentition.^37^ A clinical study^45^ with severe tooth-wear patients observed more anterior than posterior composite restoration failures. In a study with a 30-month clinical follow-up^19^ focusing on localized anterior tooth wear, bulk fractures or staining were found to be the main types of failures. The authors also observed the distinctive presence of wear facets.^19^ Another clinical study^47^ evaluating maxillary anterior teeth restored with buccal and palatal veneers reported the presence of a defective margin at the junction between the composite veneers. The occurrence of staining and marginal discoloration is also frequent in such patients,^19,32,37^ and may compromise the esthetics.

There is a growing body of research on restorations in patients with (severely) worn dentitions. Although it is still not entirely clear how anterior composite restorations in worn dentitions deteriorate over time, it is likely that a mechanical fatigue process is involved in failures,^9,26,51^ considering that these restorations are subjected to cyclic loading forces from mastication and parafunctional activities. Fatigue failures may take time to be clinically noticeable even for patients; thus, at least minor fractures are already present in the resin composite when patients return to the office.

Survival and success rates of anterior restorations in severely worn teeth may be reported by clinical studies, but the continuous process of deterioration can hardly be investigated in vivo. Although a challenge, successful in vitro reproduction of the in vivo deterioration of composite restorations observed in severe tooth-wear patients could allow better preclinical testing of restorative materials and techniques. By understanding the degradation process, clinical procedures could be improved by investigating methods to reduce the number of interventions or prolong the survival of restorations. Typical testing of dental materials may involve laboratory studies as first steps, followed by clinical trials.^11^ The process of converting basic science into practice is often called translational research, which is bidirectional: clinical practice and research should also feed information to laboratory analyses.^33^ In this sense, many in vitro methods that use loading and wear simulation devices have been developed and tested,^10,16–18,38,44^ presenting variations in chewing simulation mechanisms. Some devices apply mechanical cyclic compressive loads, with or without sliding, a method that has been shown to produce fatigue failures similar to those found clinically in tabletop ceramic restorations.^1^ Other simulators, such as the Rub&Roll device (Radboudumc; Nijmegen, The Netherlands),^39,41^ apply loads in a rolling motion in an endeavor to resemble chewing cycles. The device was recently shown to produce deterioration compatible to erosive cup-shaped lesions.^40^

Studies on oral simulation methods usually address the mechanical performance of posterior restorations,^5,12,21^ where loads are higher than in the anterior region, as are the mechanical challenges. Anterior restorations should also be investigated: their anatomy and occlusion function differently than those in the posterior region, and different restorative techniques are usually employed, including veneers.^19,37,45,47^ The aim of this study was to evaluate the ability of two chewing simulation devices to emulate in vitro the clinical deterioration observed at the incisal edges of anterior composite restorations placed in severe tooth-wear patients after up to 3.5 years of follow-up. Although different restorative protocols were used, we did not aim to analyze differences between the protocols, but rather the effect of the devices on clinically-relevant restoration types. The hypothesis was that surface deterioration aspects comparable to the clinical observations could be emulated in the laboratory, but that differences could be present between the two chewing simulation devices.

## Materials And Methods

### Study Design

**Fig 1:**
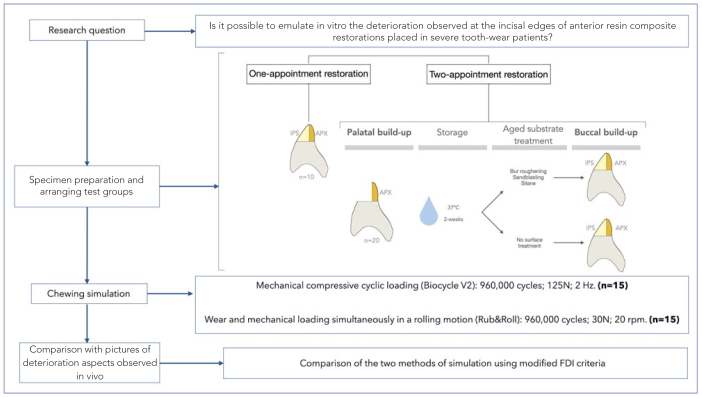
Flowchart of the study protocol (microhybrid palatal composite: Clearfil AP-X; nanohybrid buccal composite: IPS Empress Direct).

**Fig 2:**
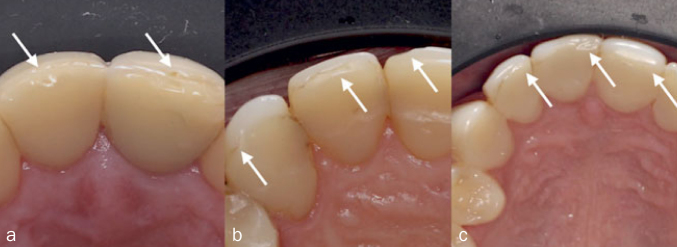
Clinical phenotypes of the incisal edges of three patients showing deterioration in their anterior direct resin composite restorations after 42 months of clinical service: (a) ditching at the incisal interface between the palatal and buccal resin composites are pointed, delamination is also visible; (b) arrows indicate wear facets and a chipping fracture at the interface, where discoloration is also visible; (c) a chipping fracture and wear facets at the interface are indicated, discoloration at the interface is also visible. (Radboud Tooth Wear Project, Radboudumc, Nijmegen, The Netherlands).^14,31^

This in vitro study compared two different chewing/oral simulation methods in terms of their ability to produce deterioration features as seen at the incisal edges of anterior resin composite restorations placed in severe tooth-wear patients within the context of the Radboud Tooth Wear project (RTWP – Radboudumc, Nijmegen, The Netherlands).^31^ One simulator applies moderate to heavy mechanical compressive cyclic loading to specimens (Biocycle V2; São Carlos, SP, Brazil), while the other combines wear and mechanical loading simultaneously in a rolling motion (Rub&Roll). Advanced tooth wear was simulated in bovine incisors, which were restored using materials (Table 1) and techniques identical to those performed in patients from the previously mentioned clinical trial,^31^ including one- or two-appointment restorations. Figure 1 presents a flowchart of the study. Specimens from different groups were restored randomly to simulate a clinical trial, and all materials were used in accordance with manufacturers’ directions. The primary response variable was the ability of the chewing simulation methods to produce surface deterioration similar to that observed clinically during recall visits at the RTWP. The most representative deterioration types are exhibited in Fig 2.

**Table 1:** Materials used in the study

Material	Brand, manufacturer	Use
Polyvinylsiloxane impression material (regular body)	Express XT, 3M Oral Care; St Paul, MN, USA	Mold created to aid restoring the palatal surface
Microhybrid resin composite	Clearfil AP-X, Kuraray Noritake; Tokyo, Japan	Palatal surface build-up
Nanohybrid resin composite	IPS Empress Direct, Ivoclar Vivadent; Schaan, Liechtenstein	Buccal surface build-up
37% phosphoric acid gel	Condac 37, FGM; Joinville, SC, Brazil	Etching of dental substrates
Priming agent	Clearfil SA Primer, Kuraray Noritake	Adhesive procedures in dental surfaces
Dual-curing bonding agent	Clearfil Photo Bond, Kuraray Noritake	
Diamond bur #4138	KG Diamond, KG Sorensen; Cotia, SP, Brazil	Roughening of palatal surface after storage
Silica-coated alumina particles (30 µm)	CoJet System, 3M Oral Care	Air abrasion of palatal surface after storage
Silane coupling agent	Clearfil Porcelain Activator, Kuraray Noritake	Chemical treatment of palatal surface after storage

### Preparation of Specimens

Thirty recently extracted bovine incisors, free of cracks and signs of wear, were collected and disinfected using 0.5% chloramine-T solution for 7 days. Advanced tooth wear was simulated by a standard flattening of the incisal third of each tooth crown using a carborundum disk under running water. Preparations involved only enamel at the incisal edges. Individual impressions using polyvinylsiloxane were taken before flattening to create a mold and facilitate the following restorative build-up process. The molds also aided in generating restorations with shape and size compatible with the original teeth.

The restorative materials and techniques were the same as those used clinically in the RTWP for direct composite build-up.^31^ Severe tooth-wear patients very often need full-mouth reconstructions, and the clinical sessions are time consuming. In the direct composite restoration protocol used in the RTWP,^31^ treatment starts with a palatal veneer build-up to re-establish the vertical dimension of occlusion. This procedure took an entire clinical appointment in some cases; thus, a second appointment was scheduled to prepare the buccal veneers. Therefore, both one- and two-session protocols were included in this study. A microhybrid composite (Clearfil AP-X, Kuraray Noritake; Tokyo, Japan) was used on the palatal surface because of its high compressive strength^15^ to withstand high-stress situations. On the buccal surface, esthetic demands are predominant and the nanohybrid composite (IPS Empress Direct, Ivoclar Vivadent; Schaan, Liechtenstein) was used because of its particle size, which can result in better polish retention and optical properties.^42^

In the restorative sequence, 37% phosphoric acid was applied to the worn surface for 20 s, after which the surface was washed thoroughly and air dried. Then a priming agent (Clearfil SA Primer, Kuraray Noritake) was rubbed on with a microbrush for 5 s and a gentle air stream was used to evaporate the solvent. Next, one drop each of the catalyst and universal liquids of an adhesive (Clearfil Photobond, Kurarary Noritake) were mixed and actively applied to the dental surface with a microbrush. A gentle air stream was applied for solvent evaporation. The bonding agent was light cured for 10 s using an LED curing unit (Radii Cal, SDI; Bayswater, Victoria, Australia) with 1200 mW/cm^2^ irradiance. The polyvinylsiloxane mold was positioned after the adhesive procedures, the palatal surface up to the incisal edge was restored using a single increment of the microhybrid composite, which was light cured for 20 s. Once the palatal surface was prepared, the specimen was randomly assigned to one of two different groups:

- One-appointment restoration (n = 10): the buccal veneer was immediately built-up using a single layer of the nanohybrid composite light cured for 20 s. No etching nor additional adhesive procedures were used;
- Two-appointment restoration (n = 20): the specimen was stored in water at 37°C for 14 days. This storage period simulated the average clinical period between two restorative sessions.^31^ Half the number of specimens in this group received the same adhesive procedure described for the palatal veneer, including acid etching, priming, and bonding. In the remaining specimens, the palatal veneer to be bonded was first roughened with a diamond bur and air abraded using silica-coated alumina particles (Cojet System, 3M Oral Care; St Paul, MN, USA). Next, the surfaces were washed thoroughly and air dried. Then, the resin composite and dental surface were etched with 37% phosphoric acid for 20 s, after which the surface was washed thoroughly and dried. A drop of silane coupling agent (Clearfil Porcelain Activator, Kuraray Noritake) was mixed with the bonding agent, according to the manufacturer’s instructions. The mixture was actively applied on the pretreated surface with a microbrush and light cured for 10 s. The nanohybrid composite was inserted and light cured.

### Chewing Simulation Methods

**Fig 3:**
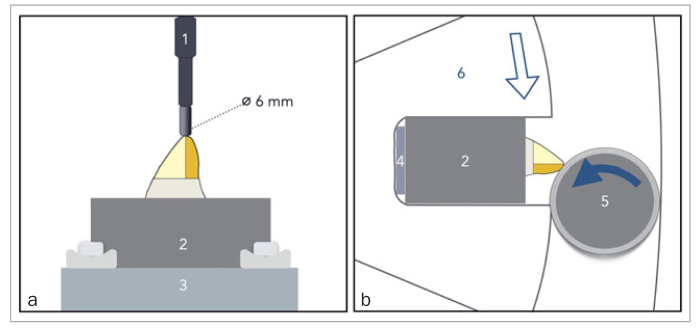
Diagrams showing the load application in the two chewing simulation devices used in this study: (a) Inside view of the container in Biocycle V2. The load piston (1) is positioned in contact with the incisal edge and perpendicular to the long axis of the specimen (2), which is inside a holder (3). An axial compressive cyclic loading is applied without impact (125 N at 2 Hz). (b) Inside view of the container in Rub&Roll. A shim (4) is placed below the specimen (2), which rotates in one direction (hollow arrow) whereas the rod (5) rotates on its own axis (filled arrow) contacting both the specimen and the rotating cylinder (6), causing wear and compression in a rolling motion (30 N load, 20 rpm).

Two different methods of chewing/oral simulation were tested (Fig 3): mechanical compressive cyclic loading applied to the center of the restored incisal edges (Biocycle V2) or a combination of wear and mechanical loading applied simultaneously in a rolling motion over the restored incisal edges (Rub&Roll).^39,41^ The methods differ mainly regarding the loading force and the contact area on surface of the specimens. Biocycle V2 applies loads to the vertical axis of the specimens by means of metallic pistons. The specimens were positioned in a metal base in individual chambers filled with deionized water at 37°C, forming a 90-degree angle between the restoration’s surface and the piston. The piston was round (6 mm diameter) and contacted only the central area of the incisal edges. A moderate axial compressive load of 125 N was applied at a frequency of 2 Hz by placing the piston in contact with the surface and then applying the load, so no abrupt impact was generated. In total, 960K cycles were performed, corresponding to about four years of aging in the mouth, which is comparable to the 3.5 years of clinical follow-up of patients whose restorations were used for comparison.^18^ In Rub&Roll, the load was applied through a loading rod placed inside a PVC tube. The specimens were individually placed in a cylinder filled with deionized water at room temperature. A 1-mm shim was positioned under each specimen to promote its protrusion. The loading rod + PVC tube rotated in the opposite direction of the cylinder containing the specimens. During each cycle, the loading rod contacted the entire incisal surface, applying a load of 30 N at 20 rpm, causing attrition at the same time.^39^ A total of 960K cycles were performed using the Rub&Roll.

### Evaluation of Restorations

An examiner (VPL) assessed clinical pictures of 240 anterior direct resin composite restorations placed in a random selection of 40 patients with severe tooth wear from the RTWP, after a period of 1 to 42 months of clinical service. Clinical pictures with signs of deterioration, as illustrated in Fig 2, were selected to allow a comparison with the deterioration generated in vitro. Restored specimens subjected to Biocycle V2 or Rub&Roll were photographed using a light stereomicroscope (Leica M50, Leica Microsystems; Wetzlar, Germany) equipped with a digital camera (Canon EOS1, Canon; Tokyo, Japan). The aged incisal surfaces were assessed by the same clinical examiner using up to 10X magnification. To compare the two simulation methods, an evaluation using modified FDI scores^20^ was performed. Four FDI clinical criteria for assessing direct restorations^20^ in terms of esthetic and functional properties were rated: surface luster, surface/marginal staining, fracture of material/retention, and marginal adaptation. The other FDI criteria were judged as not applicable to evaluate surface deterioration. For each criterion, restorations received scores between 1 and 5: scores 1–3 represent clinically acceptable restorations, whereas scores 4 and 5 are clinically unacceptable, implying the need for restoration repair or replacement, respectively. Six specimens exhibiting ditching and delamination at the composite-composite interface were selected for a more in-depth surface observation using field-emission scanning electron microscopy (FE-SEM, Zeiss Sigma 300, Carl Zeiss; Oberkochen, Germany). The smear layer resulting from loading was carefully removed by immersing the specimens in 2.5% NaOCl solution for 3 min during constant agitation with a brush.^49^ The specimens were air dried, sputter-coated with chromium in a high-vacuum evaporator, and analyzed using FE-SEM.

### Data Analysis

Signs of surface deterioration observed for the restorations aged in vitro were descriptively compared with the clinical pictures of anterior direct resin composite restorations showing deterioration in vivo (Fig 2). Data for the FDI scores were statistically analyzed considering the chewing simulation method as a factor of comparison. Differences in frequency distributions of the FDI criteria between restorations subjected to the two simulation devices were analyzed using Fisher’s exact test (α = 0.05). Statistical analysis was carried out using the SPSS v.1.0.0.1275 statistical package (Chicago, IL, USA). As the aim of this study was to evaluate the simulation devices alone, one- and two-appointment restorations or different surface treatments were not compared. FE-SEM images of the deterioration in restored incisal edges were analyzed qualitatively.

## Results

**Fig 4:**
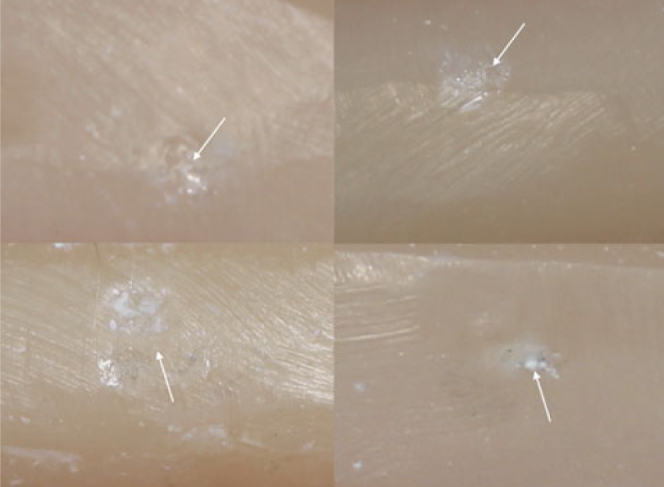
Deterioration features produced by the mechanical compressive cyclic loading (Biocycle V2) in four different specimens: minor signs of wear and contact damage (arrows) limited to the area in contact with the load piston were observed, exhibiting a typical pattern of localized rough surface. These surface deterioration features were not considered compatible with signs of deterioration observed in vivo.

**Fig 5:**
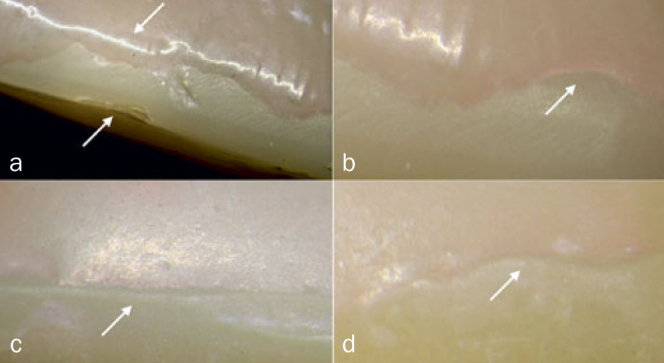
Deterioration features produced by the combination of wear and mechanical loading simultaneously in a rolling motion (Rub&Roll) in four different specimens: (a) wear facets and chipping fracture; (b) delamination and staining; (c) wear facets and marginal ditching; (d) marginal ditching. These surface deterioration features resembled those signs of deterioration found in vivo.

One specimen in the Rub&Roll group fractured prematurely and was excluded from the analysis. Signs of surface degradation were observed in restorations from both chewing-simulation groups, albeit with a remarkable difference between the deterioration patterns. Specimens subjected to mechanical compressive cyclic loading in Biocycle V2 exhibited minor signs of wear and contact damage limited to the area associated with the loading piston (Fig 4), showing a typical pattern of a localized rough surface. These surface deterioration features were not comparable to the clinical signs of deterioration. In contrast, restorations subjected to the method combining wear and mechanical loading simultaneously in a rolling motion (Rub&Roll) presented signs of surface degradation that were similar to the clinical degradation features. As shown in Fig 5, restorations subjected to the Rub&Roll showed wear facets, chipping fracture, delamination, staining, and/or marginal ditching along the incisal edges, resembling the deterioration found in vivo.

Table 2 shows the frequency distribution of the FDI criteria for restorations subjected to the different simulation methods. Regarding the esthetic properties, specimens subjected to Rub&Roll showed statistically significant differences compared to specimens subjected to Biocycle V2 for surface/marginal staining (p<0.001), whereas surface luster did not differ (p = 0.3). Regarding functional properties, for the specific criteria fracture of material/retention and marginal adaptation, specimens subjected to Rub&Roll exhibited significant differences compared to Biocycle V2 (p = 0.003). Overall, a score of 4 was given only to the specimens subjected to the Rub&Roll.

**Table 2:** FDI scores of anterior resin composite restorations subjected to the different chewing simulation methods

General criteria	Specific criteria	Biocycle V2 (n=15)	Rub&Roll (n=14)	p-value*
		Restorations within each score (1/2/3/4/5)		
Esthetic properties	Surface luster	6/9/0/0/0	6/2/6/0/0	0.3
	Surface/marginal staining	15/0/0/0/0	5/9/0/0/0	< 0.001
Functional properties	Fracture of material/retention	11/0/4/0/0	3/3/3/5/0	0.003
	Marginal adaptation	11/4/0/0/0	2/10/2/0/0	0.003

FDI scores^20^: 1. clinically excellent/very good; 2. clinically good; 3. clinically sufficient/satisfactory; 4. clinically unsatisfactory; 5. clinically poor.

*Fisher’s exact test.

**Fig 6:**
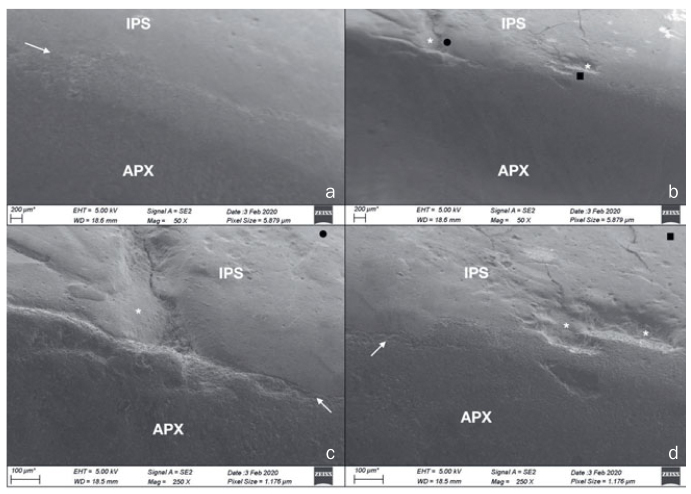
FE-SEM images (different magnifications) of a resin composite restoration subjected to Rub&Roll (APX: Clearfil AP-X; IPS: IPS Empress Direct). The arrows indicate the interface between the palatal (APX) and buccal (IPS) resin composites. Chipping fractures at the interface and extending to the buccal composite are indicated by asterisks. Black circles and squares indicate respectively the areas shown at higher magnification in c and d. In (a), a continuous, well-preserved interface between the palatal (APX) and buccal (IPS) resin composites is visible in some areas, with no gaps or voids. The palatal composite shows a rougher surface compared with the buccal composite (50X). In other areas of the specimen (b), cracks and minor chipping fractures are present (50X). Fracture at the interface between the two resin composites is observed in (c). The palatal resin composite shows minor chippings, less frequently and with lower sizes as compared with the buccal composite (250X). In (d), another fracture at the interface with microcracks is visible at the buccal side (250X).

**Fig 7:**
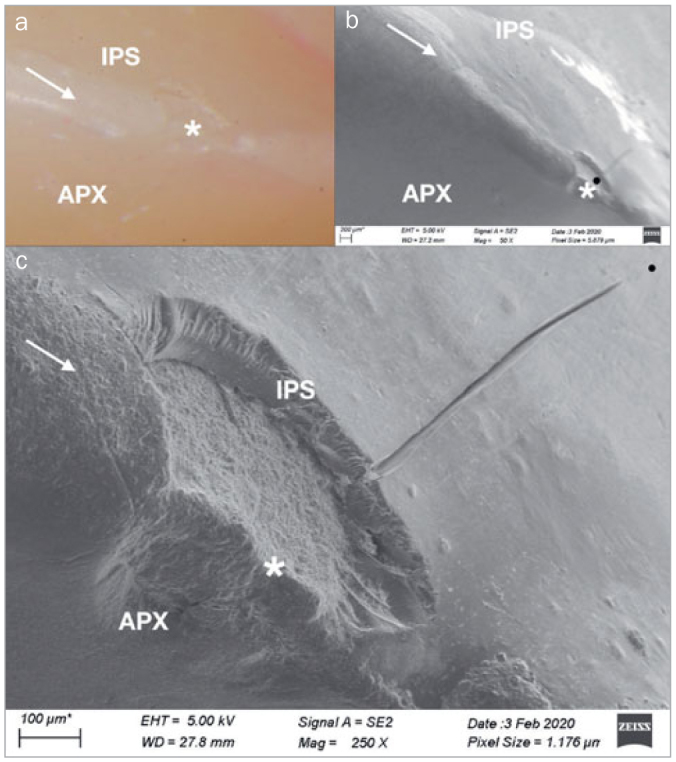
Optical microscope and FE-SEM images (different magnifications) of a resin composite restoration subjected to Rub&Roll (APX: Clearfil AP-X; IPS: IPS Empress Direct). The arrows indicate the interface between the palatal (APX) and buccal (IPS) resin composites. Fractures are indicated by asterisks. The black circle indicates the area shown at higher magnification in (c). In (a), the presence of wear facets and an adhesive fracture is visible at the interface between the palatal (APX) and buccal (IPS) resin composites (10X). In (b), a microcrack connected to the adhesive fracture is visible within the buccal resin composite (50X). In (c), a delamination effect with a hackle pattern is present on the fracture walls (250X).

Figure 6 presents FE-SEM images with different magnifications of the incisal edge of a restoration subjected to the Rub&Roll. A continuous, well-preserved interface between the palatal (Clearfil AP-X) and buccal (IPS Empress Direct) resin composites was visible in some areas, with no signs of gaps or voids (6a). The palatal composite showed a rougher surface compared with the buccal composite. In other areas of the specimen, however, cracks and minor chipping fractures were present (6b–6d). One remarkable feature is that those cracks and chippings were located at the interface between the two resin composites, and were usually present in the resin composite on the buccal side. The palatal resin composite showed signs of wear and minor chippings, but less frequently and with smaller areas than those found on the buccal composite. Figure 7 shows an optical-microscope image of another specimen subjected to the Rub&Roll, where the presence of wear facets and an adhesive fracture at the interface between the two resin composites are visible (7a). FE-SEM images of the same specimen showed a microcrack connected to the adhesive fracture within the buccal composite (7b). Higher magnification of the adhesive fracture showed delamination with a hackle pattern on the fracture walls (7c).

## Discussion

To our best knowledge, this is the first study to attempt in vitro emulation of the deterioration observed in vivo in anterior resin composite restorations placed in severe tooth-wear patients. The mechanical compressive cyclic loading method (Biocycle V2) produced deterioration features that were not comparable to those observed in vivo. In contrast, the method of combining wear and mechanical loading simultaneously in a rolling motion (Rub&Roll) produced deterioration effects resembling those observed in vivo, including wear facets, chipping fracture, delamination, staining, and/or marginal ditching along the incisal edges. Thus, the study hypothesis could not be rejected.

In the mouth, composite restorations are subjected to various conditions and challenged by a combination of different mechanisms including mechanical cycling, abrasive, attritive, and erosive processes.^26^ The complexity of this process makes it complicated to correlate the clinical and laboratory performances of resin composites.^7^ In severe tooth-wear patients, the deterioration is also not easily detected clinically, which makes it harder to establish parameters for comparison between laboratory and clinical findings. It is important to acknowledge that the correlation between clinical and laboratory findings done in this study was based on a descriptive comparison with clinical pictures, which is not an objective or quantitative method. This limitation is consistent with the fact that, to our knowledge, this is also the first study to examine the deterioration process in anterior restorations placed in severe tooth-wear patients. Considering that there is no established parameter that would allow us to perform a more direct analysis, a visual inspection and comparison with the clinical pictures seemed a reasonable method to address the research question.

The restorative technique of placing two composite veneers to reconstruct the anterior teeth, one palatal and one buccal veneer, has been described previously.^23,29,31,36,48^ As a result, the incisal edge of the restorations consisted of two layers of different resin composites forming an interface that was subjected to cyclic loading. FE-SEM images highlighted the differences between the composites after loading and wear: Clearfil AP-X showed a rougher surface than did IPS Empress Direct. This difference might be related to their distinct filler particle sizes. The nanohybrid IPS has smaller filler particles (0.55 µm average) than does the microhybrid AP-X (average 3 µm).^46^ Surface roughness of worn composites is dictated by their largest inorganic particles, because they leave larger spaces when dislodged from the surface during mechanical aging. Our observation is consistent with findings from a previous in vitro study,^22^ which showed that after a combination of occlusal and brushing wear, Clearfil AP-X had the greatest wear depth and the roughest surface texture among the materials tested.

IPS Empress Direct showed a smoother surface after aging but a higher prevalence of microcracks and chipping fractures within the composite as compared with Clearfil AP-X. This finding may be explained by the lower filler volume of IPS (52-59%) compared with AP-X (70%), as the presence of stiffer inorganic particles may increase the fracture toughness and reduce microcrack propagation in dental resin composites.^28,34^ The propagation of microcracks due to repeated occlusal loading may follow an interparticle crack path into the polymer matrix or at the boundary of filler by particle interfaces.^26^ It is likely that despite its smoother surface, a considerable number of particles of IPS Empress Direct were removed or fragmented as a result of the repetitive loading and strained the material beyond its fatigue limit. An in vitro study^25^ found behavior similar to that observed here for two nanohybrid composites with filler particle sizes similar to IPS Empress Direct, exhibiting pronounced crack formation. Other factors related to the composition of the resin composites might be involved in their response to cyclic loading, including the bond strength between filler particles and organic matrix.^26^

Fatigue failure refers to the formation and propagation of microcracks of a material due to repetitive or cyclic loading. Intraorally, teeth and restorative materials undergo cyclic loading by masticatory movements and parafunctional habits. A gradual deterioration of restorative materials by fatigue is a relevant concern because repetitive or cyclic loading might lead to subcritical microcrack propagation and ultimately fatigue fractures.^2,3^ The presence of a composite-composite interface at the incisal edge of the restored teeth represents an additional concern in the fatigue process. This interface should be of maximum quality, as voids or other irregularities present along the interface could act as stress magnifiers and initiate mechanical failure.^27^ The effect of stresses concentrated at the interface was particularly visible in specimens subjected to the Rub&Roll. During each cycle, the loading rod caused strain along a large area of the incisal edge, allowing the microcracks to grow and propagate. The broad area contacted by the loading rod, along with the low volume of restoration at the incisal edge, may have impaired stress dissipation, thus resulting in more deterioration as compared with Biocycle V2. In the latter, the load was applied at the central area of the incisal edge only, with a smaller area subjected to direct stress concentration. This could have led to less microcracking and less overall degradation, resulting in deterioration that was not comparable to that observed in vivo. Future studies could address whether a wider diameter or a different material composition of the loading piston could influence the deterioration promoted by Biocycle V2 or other similar chewing simulation devices.

A previous study using a mechanical compressive cyclic loading method similar to Biocycle V2 reported the ability to produce fatigue failures similar to those found clinically on tabletop ceramic restorations.^1^ This observation differs from our findings, which showed that the load specimens exhibited effects limited to the area in contact with the indenter. A possible explanation for these differences should consider that ceramics are more brittle and may show different failure characteristics than resin-based materials.^26^ In addition, the findings may be related to differences in loading magnitude (200–450 N vs 125 N), loading frequency (4 vs 2 Hz), and number of piston contact points (3 vs 1) between the quoted and the present study, respectively. As the aim here was to replicate oral conditions, the test frequency should not exceed 2 Hz, which is the higher range of typical human chewing frequency.^6^ A lower loading magnitude was also chosen to better reproduce the oral conditions, as physiological masticatory forces were found to be between 20 and 160 N.^8^ The method of applying wear and mechanical loading simultaneously in a rolling motion also exerted a force within this range, albeit of significantly lower magnitude compared to the compressive cyclic loading method.

The specimen geometry is another important factor to be considered when emulating clinical conditions in the laboratory. A virtue of the present study was the use of full bonded restorations as test specimens, just as they were performed in the clinical study.^31^ For that, we also simulated advanced coronal destruction seen in severe tooth-wear patients, which means that the surface subjected to loading forces consisted of restorative materials alone. This aspect provided a more comprehensive condition to evaluate the resin composites than if we had used geometrical composite specimens or non-bonded restorations.^50^ A limitation of the study is that, although clinically relevant effects were produced by the Rub&Roll, restorations are subjected to a more complex loading scenario in the oral environment, simultaneously comprising various mechanical and wear conditions.^35^ However, the primary intent was to determine if emulation of clinical deterioration was feasible in vitro as an initial step to a translational approach. To this end, we included the two different restorative-appointment groups to ensure that the procedures reflected clinically reality. The next step will be to focus on the effect of one or two restorative appointments on the deterioration, using the method of combining wear and mechanical loading simultaneously in a rolling motion. Further research is also planned to use this method focusing on the location of the interface and the role of restorative materials and composite surface treatments on the deterioration process. Although challenging, in vitro reproduction of the deterioration in resin composite restorations may be important in the preclinical testing of restorative materials and techniques. Improved laboratory tests may be useful to generate evidence that could enhance clinical procedures, reduce unnecessary interventions, and prolong the clinical survival of restorations in high-risk patients.

## Conclusions

This study shows that the in vitro method of simultaneously combining wear and mechanical loading in a rolling motion (Rub&Roll) was able to emulate in vitro the surface deterioration effects observed in anterior composite restorations placed in patients with severe tooth wear. This simulation method may contribute to translational research by allowing in vitro simulation of clinical deterioration of resin composite restorations.

## Clinical relevance:

Laboratory methods capable of emulating in vivo observations may aid in the development of preclinical tests useful for generating data that can be translated into clinical practice.
